# Tubeimoside I Protects Dopaminergic Neurons Against Inflammation-Mediated Damage in Lipopolysaccharide (LPS)-Evoked Model of Parkinson’s Disease in Rats

**DOI:** 10.3390/ijms19082242

**Published:** 2018-07-31

**Authors:** Dewei He, Bingxu Huang, Shoupeng Fu, Yuhang Li, Xin Ran, Yandan Liu, Guangxin Chen, Juxiong Liu, Dianfeng Liu

**Affiliations:** College of Animal Science and Veterinary Medicine, Jilin University, Changchun 130062, China; hedw9914@mails.jlu.edu.cn (D.H.); huangbx16@mails.jlu.edu.cn (B.H.); fushoupeng@jlu.edu.cn (S.F.); yhli9915@mails.jlu.edu.cn (Y.L.); ranxin9914@mails.jlu.edu.cn (X.R.); liuyd9915@jlu.edu.cn (Y.L.); chengx15@mails.jlu.edu.cn (G.C.)

**Keywords:** Tubeimoside I, microglia, Parkinson’s disease, MAPKs, NF-κB

## Abstract

Parkinson’s disease (PD), a frequent degenerative disease in the elderly, is characterized by dopaminergic neurodegeneration in the substantia nigra pars compacta (SNpc). Neuroinflammation caused by over-activated microglia plays a crucial role in the pathogenesis of PD. Tubeimoside I (TBMS1) has a broad anti-inflammatory effect in peripheral tissues, but the effect on neuroinflammation has not been reported. Therefore, we explored whether TBMS1 could protect dopaminergic neurons by inhibiting the activation of microglia in lipopolysaccharide (LPS)-induced PD rat model. In addition, then, the effect and mechanism of TBMS1 on neuroinflammation were assessed in LPS-exposed murine microglial BV-2 cells. The results in vivo showed that TBMS1 suppressed microglial activation and dopaminergic neurons’ reduction in LPS-injected PD rat model. In vitro study found that TBMS1 could inhibit LPS-induced inflammatory responses in BV-2 cells, and this effect was mediated by suppressing the phosphorylation of protein kinase B (AKT), nuclear factor-kappa B (NF-κB p65), p38 and extracellular regulated protein kinases (ERK1/2). Taken together, these results demonstrated for the first time that TBMS1 played a role in protecting dopaminergic neurons by inhibiting neuroinflammation mediated by microglia.

## 1. Introduction

Parkinson’s disease (PD), a common neurodegenerative disease in middle-aged and elderly people, is characterized by selective loss of dopaminergic neurons in the substantia nigra pars compacta (SNpc) region of the midbrain [1]. The major clinical symptoms of PD are resting tremor, rigidity and bradykinesia [2]. Accumulating studies suggest that the pathogenesis of PD is related to several factors, including environmental and genetic factors, oxidative stress, and mitochondrial dysfunction. However, evidence has indicated that microglia-mediated inflammation may play an important role in the neurodegenerative process. McGeer et al. for the first time demonstrated the presence of activated microglia in the substantia nigra (SN) of PD brains [3]. Since then, a lot of studies have found that microglia-mediated neuroinflammation contributes to the pathogenesis of several neurodegenerative disorders including PD [4,5]. A large amount of evidence has revealed that activated microglia can produce various neurotoxic factors, including proinflammatory cytokines (interleukin-1β (IL-1β), interleukin-6 (IL-6), tumor necrosis factor-α (TNF-α)) and metabolites (prostaglandin E2 (PGE2) and nitric oxide (NO)) of proinflammatory enzymes, which can damage the surrounding dopaminergic neurons [6,7,8]. Thus, therapeutic intervention that inhibits neuroinflammation incurred by activated microglia would be an effective therapy in PD.

Lipopolysaccharide (LPS) is an endotoxin found in the cell walls of Gram-negative bacteria and is a potent stimulator of microglia. It could induce the activation of microglia in vitro and in vivo. Thus, LPS is widely used as a neuroinflammation elicitor to generate cellular/animal phenotypes of PD [9,10,11]. In addition, the LPS-injected PD rat model and the LPS-induced microglial inflammatory responses have been widely used to study the anti-inflammatory mechanism of some substances in vitro and in vivo [12,13,14].

Bolbostemma, a traditional herbal medicine, has antiviral and anticancer effects [15,16,17]. As a natural plant tuber, Bolbostemma is widely used in the pharmaceutical industry. Tubeimoside I (TBMS1) extracted from Bolbostemma has been reported to attenuate inflammation and oxidative damage in a mice model of pulmonary injury [18] and inhibit synoviocytes and collagen-induced arthritis in rats [19]. Moreover, numerous studies have demonstrated that TBMS1 exerted anti-inflammation and inhibition of the cancer cells proliferation in peripheral tissues [20,21,22]. However, no reports have shown whether TBMS1 has an anti-inflammatory effect in the central nervous system. Therefore, in the present study, we investigated the neuroprotective and anti-inflammatory properties of TBMS1 in PD rat model and elucidated its anti-inflammatory mechanism in LPS-exposed BV-2 cells. Our results showed that TBMS1 prevented the loss of dopaminergic neurons by inhibiting microglial activation in PD rat model. In addition, the mechanistic study revealed that the inhibitory effect of TBMS1 on microglial activation was mediated by suppressing the phosphorylation of AKT, NF-κB p65, p38 and ERK1/2. Based on these findings, we suggest an additional potential tool for the therapeutic intervention of PD.

## 2. Results

### 2.1. TBMS1 Treatment Improves the Behavioral Dysfunction of LPS-Injected PD Rat Model

**Figure 1:**
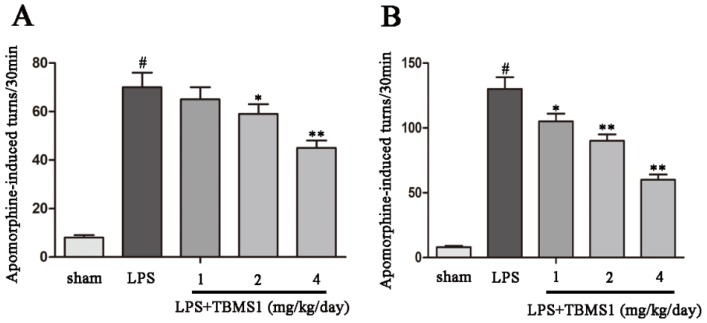
Tubeimoside I (TBMS1) improves the behavioral dysfunction of lipopolysaccharide (LPS)-induced Parkinson’s disease (PD) rat model. The rats were pretreated with TBMS1 (1, 2, 4 mg/kg/day) for three days before LPS injection and, subsequently, for 21 days after LPS injection. The number of turns induced by amphetamine were counted at two (**A**) and four weeks (**B**). ^#^ *p* < 0.01 versus the sham-operated group. * *p* < 0.05 and ** *p* < 0.01 versus the TBMS1-untreated LPS-injected group, and statistical significance was determined by ANOVA.

Injection of LPS into the unilateral SN results in microglial activation and damage to dopaminergic neurons. Unilateral injury of dopaminergic neurons results in excessive activation of compensatory dopamine receptors, which leads to the rotation behavior towards the injection side. To examine the effect of TBMS1 on motor dysfunction, the rotational behaviors of LPS-injected PD rat model were detected at the second and fourth weeks after the beginning of TBMS1 treatment. The results showed that TBMS1 treatment obviously decreased amphetamine-induced rotational behaviors in the LPS-injected PD rat model, and the role of TBMS1 in the fourth week is more effective than the second week (Figure 1).

### 2.2. TBMS1 Treatment Protects Dopaminergic Neurons in the SN

**Figure 2:**
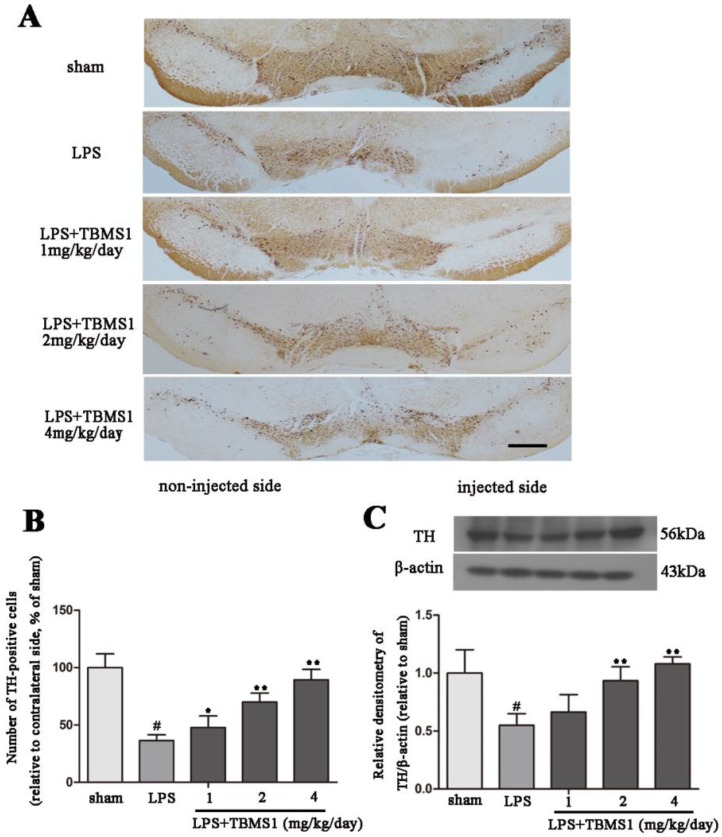
Protective effects of TBMS1 on dopaminergic neurons in the substantia nigra (SN) in LPS-injected PD rat model. PD rat models were anesthetized and sacrificed to obtain the midbrain after 4 weeks’ TBMS1 treatment. (**A**) Tyrosine hydroxylase (TH)-positive cells in 40× Fluorescence microscope via immunohistochemistry, the scale bar represents 100 μm (*n* = 5 per group); (**B**) TH-positive cells number (TH-positive cells on the injected side versus the TH-positive cells on non-injected side) was blind-counted by five individuals; (**C**) The protein level of TH was tested by western blotting. The results are shown as mean ± SD of three independent experiments. ^#^ *p* < 0.01 versus the sham-operated group. * *p* < 0.05 and ** *p* < 0.01 versus the TBMS1-untreated LPS-injected group, and statistical significance was determined by ANOVA.

It has been confirmed that dopaminergic neurons are damaged in the course of PD. To explore the protective effect of TBMS1 on dopaminergic neurons, we took the midbrain of rats, sliced and processed. Then the number of tyrosine hydroxylase (TH)-positive cells was detected by immunohistochemistry and the protein level of TH protein was detected by western blotting. Immunohistochemistry results showed that in the sham-operated group, the number of TH-positive cells was equal on the non-injected side and the injected side, which means that surgical operation did not affect the survival of dopaminergic neurons (Figure 2A). Compared with the sham-operated group, the number of TH-positive cells decreased prominently in the LPS group (Figure 2A,B). In addition, TBMS1 treatment significantly inhibited the loss of dopaminergic neurons in a dose-dependent manner in LPS-injected PD rat model. The result of TH protein level was similar to the trend of the change of TH-positive cells number, and TBMS1 could inhibit LPS-induced decline of TH protein level (Figure 2C). These experimental results suggest that TBMS1 could prevent the loss of dopaminergic neurons in LPS-injected PD rat model.

### 2.3. TBMS1 Inhibits LPS-Induced Over-Activation of Microglia in the SN

**Figure 3:**
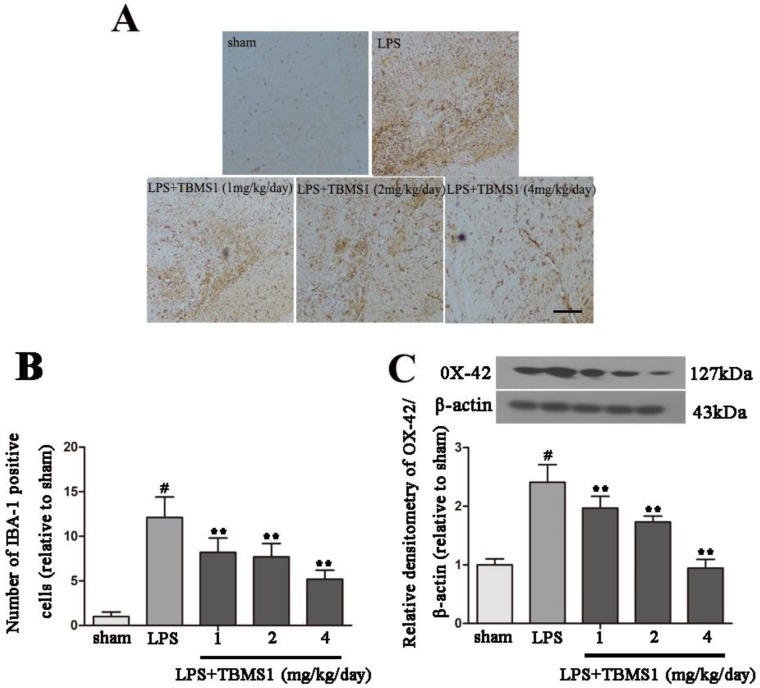
Effect of TBMS1 on microglial activation. The PD rat models were anesthetized and sacrificed to obtain the midbrain after 4 weeks’ TBMS1 treatment. (**A**) Calcium-binding adaptor molecule-1 (IBA-1)-positive cells in 100 × Fluorescence microscope via immunohistochemistry, the scale bar represents 200 μm (*n* = 5 per group); (**B**) IBA-1-positive cells’ number was counted by five individuals without disturbing each other; (**C**) The protein level of OX-42 was tested by western blotting. The result was shown as mean ± SD of five independent experiments. ^#^ *p* < 0.01 versus the sham-operated group. ** *p* < 0.01 versus the TBMS1-untreated LPS-injected group, and statistical significance was determined by ANOVA.

Numerous studies have confirmed that neuroinflammation mediated by over-activated microglia plays an important role in the development of PD [23,24,25]. Based on previous studies, we evaluated whether the inhibition of microglial activation was associated with the neuroprotective effect of TBMS1. Ionized calcium binding adaptor molecule-1 (IBA-1), a specific marker for microglial activation, was examined by immunohistochemistry. Consistent with other reports, microglia transform from ramified resting cells to an activated amoeboid morphology after LPS injection (Figure 3A), and TBMS1 treatment dramatically suppressed the activation and decreased the number of microglia in a dose-dependent manner (Figure 3A,B). OX-42 is another marker for microglial activation; to obtain quantitative data, we examined the protein level of OX-42 in the SN. The results showed that, compared with sham-operated rats, intranigral injection of LPS significantly increased the protein level of OX-42 (Figure 3C). In addition, TBMS1 treatment dose-dependently decreased the protein level of OX-42 in LPS-injected PD rat model (Figure 3C). These data suggest that inhibition of microglial activation is one of the mechanisms that TBMS1 protects dopaminergic neurons.

### 2.4. Effect of TBMS1 on the Viability of BV-2 Cells

**Figure 4:**
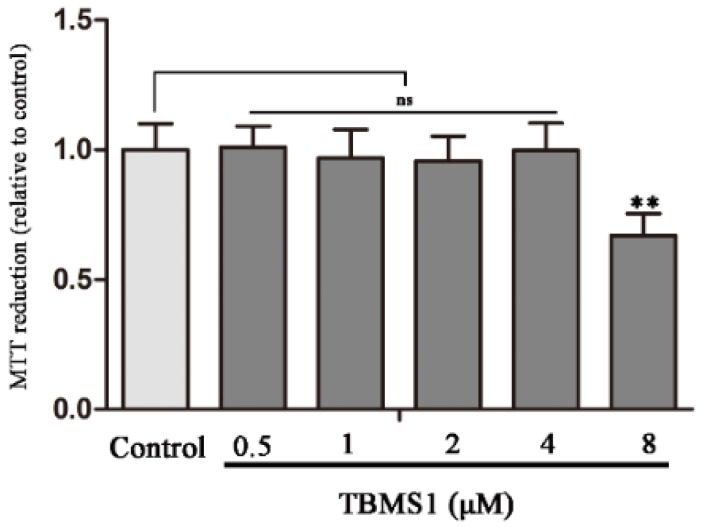
Effect of TBMS1 on the viability of BV-2 cells. The BV-2 cells were incubated for 24 h with TBMS1 (0–8 μM) and then the viability of BV-2 cells was detected using MTT assay. The results are shown as mean ± SD of five independent experiments. ** *p* < 0.01 versus control condition, and statistical significance was determined by ANOVA.

To determine the potential cytotoxicity of TBMS1, the viability of BV-2 cells was measured by MTT assay. After exposure of BV-2 cells to different concentrations of TBMS1 for 24 h, the cells were collected to evaluate the cytotoxicity of TBMS1. We found that TBMS1 for 0–4 μM did not affect the viability of BV-2 cells, but TBMS1 for 8 μM notably reduced the viability of BV-2 cells (Figure 4).

### 2.5. TBMS1 Inhibits the Production of Pro-Inflammatory Cytokines in LPS-Exposed BV-2 Cells

**Figure 5:**
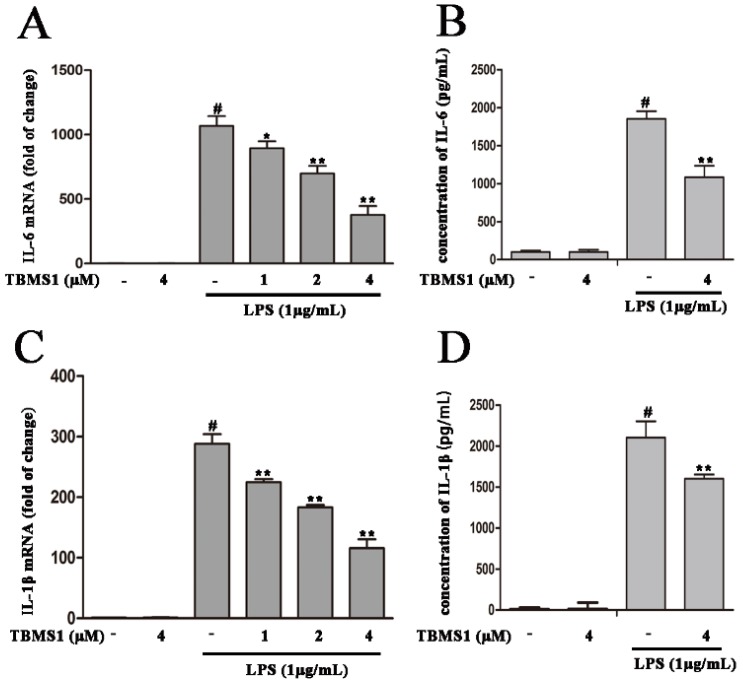

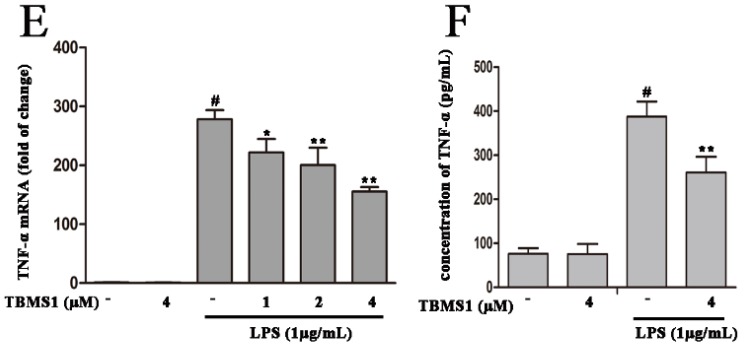
Effects of TBMS1 on the mRNA and protein levels of pro-inflammatory cytokines in LPS-exposed BV-2 cells. The BV-2 cells were pretreated with TBMS1 for 1 h, and then incubated with LPS (1 μg/mL) for 4 h (mRNA) or 24 h (protein). The mRNA levels of interleukin-6 (IL-6) (**A**), interleukin-1β (IL-1β) (**C**) and tumor necrosis factor-α (TNF-α) (**E**) in BV-2 cells were examined by quantitative real-time PCR. The relative mRNA levels of TNF-α, IL-1β and IL-6 were calculated relative to β-actin. The protein levels of IL-6 (**B**), IL-1β (**D**) and TNF-α (**F**) were examined by ELISA. The results are shown as mean ± SD of three independent experiments. ^#^ *p* < 0.01 versus control condition. * *p* < 0.05 and ** *p* < 0.01 versus the TBMS1-untreated LPS-exposed cells, and statistical significance was determined by ANOVA.

Pro-inflammatory cytokines (IL-6, IL-1β and TNF-α) produced by activated microglia play a vital role in degeneration of dopaminergic neurons in the SN. To explore whether TBMS1 suppresses the production of IL-6, IL-1β and TNF-α, firstly, BV-2 cells were treated with TBMS1 (1, 2 and 4 μM) for 1 h then stimulated with LPS (1 µg/mL) for 4 h, the mRNA levels of IL-6, IL-1β and TNF-α were detected by quantitative real-time PCR. The results showed that LPS markedly increased the mRNA levels of IL-6, IL-1β and TNF-α, and TBMS1 was able to significantly inhibit this effect in a concentration-dependent manner (Figure 5A,C,E). Next, BV-2 cells were treated with TBMS1 (4 μM) for 1 h and then stimulated with LPS (1 µg/mL) for 24 h, and the effects of TBMS1 on the protein levels of IL-6, IL-1β and TNF-α in media supernatants were examined by ELISA. The results showed that TBMS1 markedly inhibited LPS-induced protein levels of IL-6, IL-1β and TNF-α (Figure 5B,D,F).

### 2.6. TBMS1 Inhibits the Production of Pro-Inflammatory Enzymes in LPS-Exposed BV-2 Cells

**Figure 6:**
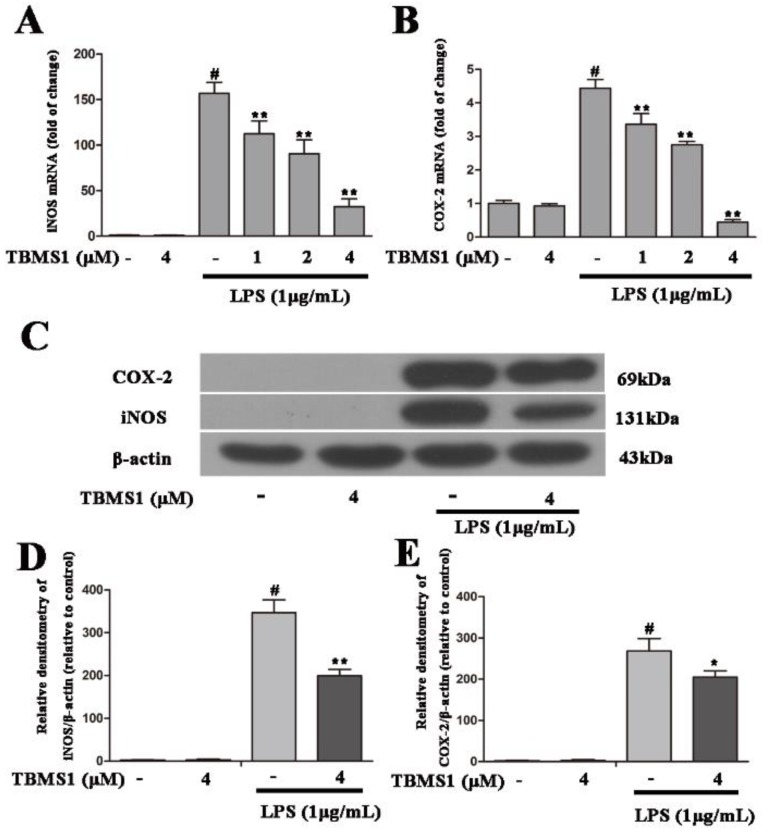
Effects of TBMS1 on the production of pro-inflammatory enzymes in LPS-exposed BV-2 cells. The mRNA levels of inducible nitric oxide synthase (iNOS) (**A**) and cyclooxygenase-2 (COX-2) (B) in BV-2 cells were examined by quantitative real-time PCR. The relative mRNA levels of iNOS and COX-2 were calculated relative to β-actin. The BV-2 cells were treated for 1 h with TBMS1 (1, 2 and 4 μM), then incubated with LPS (1 μg/mL) for 4 h. The protein levels of iNOS (**C**,**D**) and COX-2 (**C**,**E**) in BV-2 cells were tested via western blotting. The BV-2 cells were treated for 1 h with TBMS1 (4 μM), then incubated with LPS (1 μg/mL) for 24 h. The results are shown as mean ± SD of three independent experiments. ^#^ *p* < 0.01 versus control condition. * *p* < 0.05 and ** *p* < 0.01 versus the TBMS1-untreated LPS-exposed cells, and statistical significance was determined by ANOVA.

Pro-inflammatory enzymes (iNOS and COX-2) are involved in LPS-induced neuroinflammation. To explore whether TBMS1 inhibits the production of iNOS and COX-2, firstly, BV-2 cells were treated with TBMS1 (1, 2 and 4 μM) for 1 h and then stimulated with LPS (1 µg/mL) for 4 h, and the mRNA levels of iNOS and COX-2 were detected by quantitative real-time PCR. The results showed that LPS markedly increased the mRNA levels of iNOS and COX-2, but TBMS1 was able to significantly inhibit this effect in a concentration-dependent manner (Figure 6A,B). Next, BV-2 cells were treated with TBMS1 (4 μM) for 1 h and then stimulated with LPS (1 µg/mL) for 24 h, and the effects of TBMS1 on protein levels of iNOS and COX-2 were examined by western blotting. The results showed that TBMS1 markedly inhibited LPS-induced protein levels of iNOS and COX-2 (Figure 6C–E).

### 2.7. TBMS1 Inhibits the Phosphorylation of AKT, NF-κB p65, ERK1/2 and p38 in LPS-Exposed BV-2 Cells

**Figure 7:**
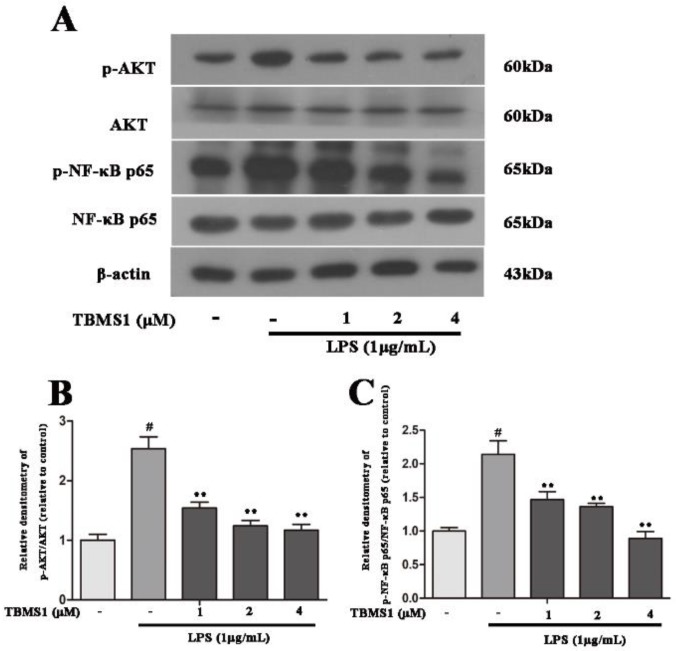
TBMS1 inhibits the phosphorylation of AKT and NF-κB p65 in LPS-exposed BV-2 cells. The BV-2 cells were pretreated with TBMS1 (1, 2 and 4 μM) for 1 h, then incubated with LPS (1 μg/mL) for 1 h. (**A**) The protein levels of AKT, NF-κB p65 and their phosphorylated forms were tested by western blotting. The phosphorylation of AKT (**B**) and NF-κB p65 (**C**) was analyzed relative to β-actin. The results are shown as mean ± SD of three independent experiments. ^#^ *p* < 0.01 versus control condition. ** *p* < 0.01 versus the TBMS1-untreated LPS-exposed cells, and statistical significance was determined by ANOVA.

**Figure 8:**
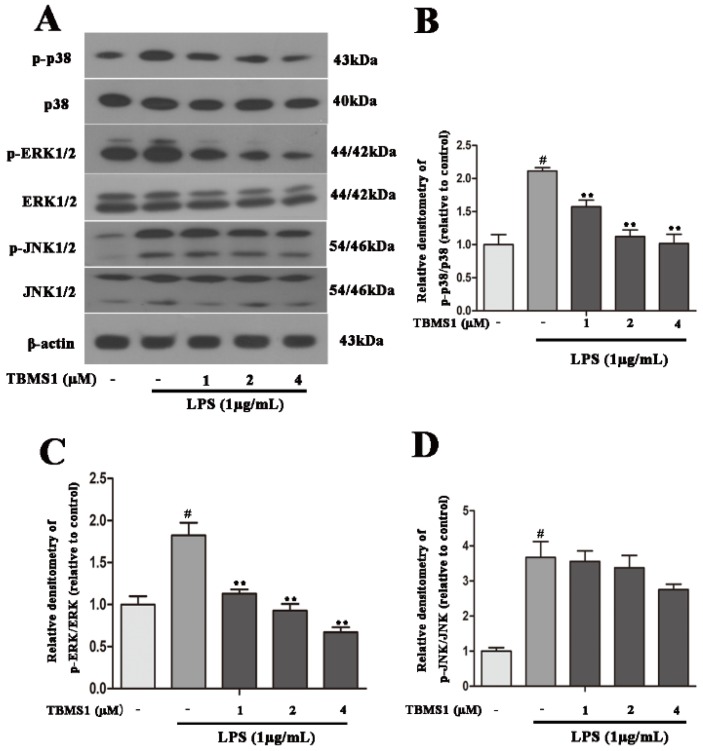
TBMS1 inhibits the phosphorylation of p38 and ERK1/2 in LPS-exposed BV-2 cells. The BV-2 cells were pretreated for 1 h with TBMS1 (1, 2 and 4 μM), then incubated with LPS (1 μg/mL) for 1 h. (**A**) The protein levels of p38, ERK1/2, JNK1/2 and their phosphorylated forms were tested by western blotting. The phosphorylation of p38 (**B**), ERK1/2 (**C**) and JNK1/2 (**D**) was analyzed relative to β-actin. The results are shown as mean ± SD of three independent experiments. ^#^ *p* < 0.01 versus control condition. ** *p* < 0.01 versus the TBMS1-untreated LPS-exposed cells, and statistical significance was determined by ANOVA.

A large number of studies have demonstrated that AKT, NF-κB and mitogen-activated protein kinases (MAPKs) play an important role in regulating the production of pro-inflammatory mediators in activated microglia [26,27,28]. In order to clarify the anti-inflammatory mechanism of TBMS1, we investigated the effects of TBMS1 on LPS-induced phosphorylation of NF-κB p65, AKT, p38, ERK1/2 and JNK1/2 in BV-2 cells. The BV-2 cells were treated with TBMS1 of different concentrations for 1 h and then stimulated with LPS for 1 h. The results of western blotting showed that TBMS1 was able to inhibit LPS-induced phosphorylation of AKT, NF-κB p65, p38 and ERK1/2 in BV-2 cells, but had no effect on the phosphorylation of JNK1/2 (Figure 7 and Figure 8).

## 3. Discussion

In this study, we found that TBMS1 was able to improve the behavioral dysfunction and could protect dopaminergic neurons via suppressing inflammatory responses mediated by activated microglia in LPS-injected PD rat model. Further, the mechanistic studies found that TBMS1 suppressed pro-inflammatory mediators (iNOS, COX-2, TNF-α, IL-1β and IL-6) produced by activated microglia via inhibiting the phosphorylation of AKT, NF-κB p65, p38 and ERK1/2. 

A large number of studies have shown that neuroinflammation plays an important role in the development of Parkinson’s disease [25,29,30]. Microglia are the resident immune cells in the brain, and are a major participant in the process of neuroinflammation. Autopsy studies have shown that there are a large number of activated microglia in the SN of PD patients, especially in areas with large neurodegeneration (ventral and lateral parts of the substantia nigra) [31]. The cytotoxic factors produced by activated microglia could damage the surrounding neurons and finally result in neurodegenerative disease [32]. Therefore, inhibiting the activation of microglia is a potential therapy to prevent the further progression of PD. LPS is an endotoxin from gram-negative bacteria, and it is a natural activator of microglia. Injecting LPS into the unilateral SNpc of rats could lead to microglial over-activation, which selectively induces lasting degeneration of dopaminergic neurons, and these pathological changes are similar to Parkinson’s disease [33]. This PD rat model has been widely used to explore drug discovery, and a variety of agents, such as Licochalone A, Galangin and Peiminine, have been evaluated for their potential neuroprotective effects in this model [12,13,14]. TBMS1 extracted from Bolbostemma has been reported to attenuate inflammation in peripheral tissues [20]. In order to study whether TBMS1 has an anti-inflammatory role in PD, we examined the effect of TBMS1 on LPS-injected PD rat model. The rotational behavior is a common behavioral detection method in PD animal model. We found the rotation number of LPS-injected group was significantly higher than the rotation number of sham-operated group. In addition, TBMS1 treatment could inhibit amphetamine-induced rotational behavior in LPS-injected PD rat model. The results suggested that TBMS1 could improve the movement disorder of PD rats. The effect on dopaminergic neurons in the SN of PD animal model has often been used to measure the therapeutic effect of drugs. To detect the protective effect of TBMS1 on dopaminergic neurons, we investigated the number of TH-positive cells using immunohistochemistry and the expression of TH using western blotting. We found that TBMS1 treatment reduced the loss of TH-positive cells in LPS-injected PD rat model. Neuroinflammation is closely related to the pathogenesis of PD, and activation of microglia could trigger neuroinflammation, leading to dopaminergic neuron death. Based on the connection between dopaminergic neuron death and microglial activation, we measured the activation markers (IBA-1 and OX-42) of microglia using immunohistochemistry and western blotting. We found that TBMS1 treatment inhibited the activation of microglia in the SN of LPS-injected PD rat model. Taken together, our results suggest that TBMS1 ameliorated behavioral dysfunction and protected dopaminergic neurons by inhibiting microglial activation in LPS-injected PD rat model.

IL-6, IL-1β and TNF-α, which are released by microglia during neuroinflammation, play a central role in initiating degeneration of surrounding dopaminergic neurons [34]. COX-2 and iNOS are two main pro-inflammatory enzymes produced by activated microglia. COX-2 and iNOS are also involved in the process of neuroinflammation, and inhibiting the activities of COX-2 and iNOS can suppress neuroinflammation mediated by microglia and protect neurons [35,36]. To explore the effect of TBMS1 on microglial activation, the production of pro-inflammatory mediators (TNF-α, IL-1β, IL-6, iNOS and COX-2) were measured. Results showed that LPS induced the production of pro-inflammatory mediators (TNF-α, IL-1β, IL-6, iNOS and COX-2) in BV-2 cells. In addition, pretreatment with TBMS1 significantly inhibited the production of pro-inflammatory mediators (TNF-α, IL-1β, IL-6, iNOS and COX-2) in LPS-exposed BV-2 cells. Multiple signaling pathways are related to inflammatory responses of activated microglia. It has been reported that the activation of the AKT, MAPKs (p38, ERK1/2, and JNK1/2) and NF-κB signaling pathways could increase the production of pro-inflammatory mediators (IL-6, IL-1β, TNF-α, COX-2 and iNOS) [37]. To determine the anti-inflammatory mechanisms of TBMS1, the effects of TBMS1 on the activation of AKT, NF-κB and MAPKs signaling pathways were investigated using western blotting in this study. We found that TBMS1 pretreatment prominently suppressed the phosphorylation of AKT, NF-κB p65, p38 and ERK1/2 in LPS-exposed BV-2 cells. These data suggested that TBMS1 inhibited microglial inflammatory responses via suppressing AKT, NF-κB, p38 and ERK1/2 signaling pathways.

In summary, our results revealed that TBMS1 treatment prevents dopaminergic neurodegeneration by inhibiting microglia-mediated neuroinflammation. The potential anti-inflammatory mechanism of TBMS1 is inhibiting the phosphorylation of AKT, NF-κB p65, p38 and ERK1/2 in LPS-exposed BV-2 cells. Overall, these results indicate that TBMS1 has a notably neuroprotective property, suggesting that TBMS1 may serve as a potential therapeutic agent for the treatment of PD in the future.

## 4. Materials and Methods 

### 4.1. Animals and Treatment

Fifty adult male Wistar rats (290 to 320 g) were purchased from Liaoning Changsheng Biotechnology Company Limited. Our animal experiments were performed according to the experimental practices and standards made by the Institutional Animal Care and Use Committee of Jilin University (approved on 27 February 2015, Permit Number: 2015047). The LPS-injected PD rat model was established as per our previous description [38]. Briefly, the rats were injected with 2 µL LPS (5 µg/µL) or an equal volume of PBS to the right SNpc (anteroposterior (AP) 5.2 mm, lateral (LAT) 2.1 mm and dorsoventral (DV) 7.8 mm) after anesthesia. The rats were randomly divided into five groups, namely, the sham-operated group (which was injected PBS into the right SNpc), LPS-injected and treated with vehicle, LPS-injected and treated with 1, 2 or 4 mg/kg TBMS1 (>98% purity; Yuan ye Biotech, Shanghai, China) (dissolved in PBS). TBMS1 was diluted with PBS and injected intraperitoneally once daily. In addition, the rats received TBMS1 at 3 days prior to surgery and for 24 days totally. 

### 4.2. Rotational Behavior Experiment

To evaluate the degree of damage, the rats were intraperitoneally injected with amphetamine to induce rotational behavior at the second and fourth weeks after the beginning of TBMS1 treatment. The rotational behavior experiment was manipulated as previously described [38]. In brief, the rats were placed in a specific test environment to adapt a period of time. Then the number of turns in thirty minutes was recorded five minutes after amphetamine-injection.

### 4.3. TH and IBA-1 Immunohistological Analysis

After separation, the rats’ midbrains were fixed in 4% paraformaldehyde for 24 h at 4 °C, and then processed using the following procedure: alcohol (70%) for 1 h, alcohol (80%) for 1 h, alcohol (90%) for 1 h, alcohol (100%) for 1.5 h, alcohol (100%) for 1.5 h, Xylene for 30 min, dipping wax for 1 h. Then, midbrains were embedded in paraffin and sectioned (3 μm per slice). The immunohistochemistry process was performed with an Ultra-SensitiveTM S-P kit (contains endogenous peroxidase blocking solution, sheep serum, biotin-labeled goat anti-rat secondary antibody, and streptavidin-peroxidase) (MBX Biotechnologies, Fuzhou, China). The dopaminergic neurons were labeled with the anti-TH antibody (Abcam, Cambridge, UK, Diluted with 5% fetal bovine serum), and the microglia was labeled with the anti-IBA-1 antibody (Abcam, Cambridge, UK, Diluted with 5% fetal bovine serum). The experimental procedures were manipulated as previously described [39]. To determine TH- and IBA-1-positive cell numbers, the cells were respectively quantified by five researchers who had absolutely no hand in the experimental treatments.

### 4.4. Murine Microglial BV-2 Cell Cultures and Treatments

BV-2 cell line was provided by Xianzhu Xia (Institute of Military Veterinary, Academy of Military Medical Sciences, Changchun, China). BV-2 cells were cultured in DMEM high glucose complete medium or DMEM high-glucose complete medium without phenol red (Gibco, Grand Island, NY, USA), which contained 10% FBS (Gibco, Grand Island, NY, USA) and 1% penicillin-streptomycin (Invitrogen, Carlsbad, CA, USA). DMEM high-glucose medium without phenol red was used for MTT assay and ELISA assay. The complete medium was changed three times every week. The cells were passaged to 6-cm cell culture dish, 24- or 96-well plate, when the confluence of the BV-2 cells reached 80%. The cells were pretreated with 1, 2 or 4 μM TBMS1 for 1 h and then stimulated with 1 μg/mL LPS (from Escherichia coli, O55:B55) (Sigma-Aldrich, St. Louis, MO, USA) for 1, 4 or 24 h.

### 4.5. Cell Viability

The effect of TBMS1 on cell viability was detected by MTT assay. Briefly, BV-2 cells (1.5 × 10^3^/well) were seeded in 96-well plate. After the cells were cultured for 24 h at 37 °C in a humidified incubator under 5% CO_2_. Then, different concentrations of TBMS1 (0–8 μM) were added to the cultures and incubated for 24 h. 20 μL MTT (5 mg/mL) were added to each well and incubated for 4 h. After that, the supernatant was abandoned and Dimethyl sulfoxide (DMSO) (Sigma-Aldrich, St. Louis, MO, USA) (150 μL/well) was added to each well. Finally, the absorbance was measured with a microplate reader at 570 nm.

### 4.6. RNA Extraction and Quantitative Real-Time PCR

Total RNA of BV-2 cells was obtained with the Trizol reagent (Sigma-Aldrich, St. Louis, MO, USA) according to the manufacturer’s protocol. After detection of RNA concentration, the PrimeScript^®^ 1st Strand cDNA Synthesis Kit (Takara Biotechnology, Ltd., Kyoto, Japan) was used to reverse-transcribe (RT) 2 μg RNA into cDNA. The mRNA levels of iNOS, COX-2, TNF-α, IL-1β and IL-6 were measured with QuantiTect SYBR Green RT-PCR Kit (Roche, South San Francisco, CA, USA), and each sample was detected three times. The relative mRNA levels of iNOS, COX-2, TNF-α, IL-1β and IL-6 were calculated relative to β-actin using the 2^−∆∆*C*t^ method. The primer sequences of iNOS, COX-2, TNF-α, IL-1β and IL-6 are shown in Table 1.

### 4.7. Western Blotting Analysis

The midbrains of rats and BV-2 cells were obtained and homogenized in lysis buffer (Beyotime Inst. Biotech, Beijing, China) containing 20 mM Tris (pH7.5), 150 mM NaCl, 1% Triton X-100, sodium pyrophosphate, β-glycerophosphate, EDTA, Na_3_VO_4_ and leupeptin. Homogenates were centrifuged at 13,000 rpm for 20 min at 4 °C and supernatants were collected for western blotting analysis. 50 µg protein was subjected to 12% or 10% SDS-polyacrylamide gel and transferred to polyvinylidene difluoride membranes (Millipore, ON, Canada; Bedford, MA, USA). After blocking with 5% skim milk, the polyvinylidene difluoride membranes were incubated with primary antibodies against TH (1:1000) (Abcam, Cambridge, UK), OX-42 (1:1000) (Abcam), COX-2 (1:2000) (Abcam), iNOS (1:2000) (Abcam), phospho-AKT (1:2000) (Cell Signaling Technology, MA, USA), phospho-JNK1/2 (1:2000) (Cell Signaling Technology), phospho-ERK1/2 (1:2000) (Cell Signaling Technology), phospho-p38 (1:2,000), phospho-NF-κB p65 (1:2000), JNK1/2 (1:2000), ERK1/2 (1:22,000) (Cell Signaling Technology), p38 (1:2000) (Cell Signaling Technology), AKT (1:2000) (Cell Signaling Technology), NF-κB p65 (1:2000) (Cell Signaling Technology) and β-actin (1:10,000) (Santa Cruz, CA, USA). Then the polyvinylidene difluoride membranes were incubated at room temperature for 1 h against the secondary antibodies goat anti-rabbit (1:2000) or goat anti-mouse (1:2000). The proteins were tested using ECL western blotting Detection Reagents (Amersham Pharmacia Biotech, Tokyo, Japan).

### 4.8. Enzyme-Linked Immunosorbent Assay (ELISA)

BV-2 cells were seeded into 24-well plates (2 × 10^5^ cells/well). After incubation with TBMS1 for 1 h, the cells were treated with LPS (1 μg/mL) for 24 h. The protein levels of IL-6, IL-1β and TNF-α in the cell culture supernatants were measured using ELISA kits (R&D Systems, Abingdon, UK).

### 4.9. Statistical Analysis

All results are shown with mean ± SD, and processed with SPSS 12.0 statistical software package (SPSS Inc., Chicago, IL, USA). The differences between the groups were evaluated with one-way analysis of variance (ANOVA). *p <* 0.05 was considered to be statistically significant.

## Figures and Tables

**Table 1:** The primers sequences of *β-actin*, *iNOS*, *COX-2*, *TNF-α*, *IL-1β* and *IL-6*.

Gene	Sequences	Length (bp)
*β-actin*	(F) 5′-GTCAGGTCATCACTATCGGCAAT-3′	147
	(R) 5′-AGAGGTCTTTACGGATGTCAACGT-3′	
*iNOS*	(F) 5′-GAACTGTAGCACAGCACAGGAAAT-3′	158
	(R) 5′-CGTACCGGATGAGCTGTGAAT-3′	
*COX-2*	(F) 5′-CAGTTTATGTTGTCTGTCCAGAGTTTC-3′	127
	(R) 5′-CCAGCACTTCACCCATCAGTT-3′	
*TNF-α*	(F) 5′-CCCCAAAGGGATGAGAAGTTC-3′	136
	(R) 5′-CCTCCACTTGGTGGTTTGCT-3′	
*IL-1β*	(F) 5′-GTTCCCATTAGACAACTGCACTACAG-3′	139
	(R) 5′-GTCGTTGCTTGGTTCTCCTTGTA-3′	
*IL-6*	(F) 5′-CCAGAAACCGCTATGAAGTTCC-3′	138
	(R) 5′-GTTGGGAGTGGTATCCTCTGTGA-3′	

## Abbreviations

TBMS1	Tubeimoside I
PD	Parkinson’s disease
LPS	Lipopolysaccharide
SNpc	Substantia nigra pars compacta
TH	Tyrosine hydroxylase
IBA-1	Ionized calcium-binding adaptor molecule-1
